# Supplementary material for: Estimating the time-varying reproduction number of COVID-19 with a state-space method
Source: PLoS Comput Biol. 2021 Jan 29;17(1):e1008679. doi: 10.1371/journal.pcbi.1008679 (PMC7875393; doi:10.1371/journal.pcbi.1008679)
Supplement: Attachment — Submitted filename: report_koyama_horie_shinomoto.pdf [file pcbi.1008679.s001.pdf]

Report on ”Estimating the time-varying reproduction number of COVID 19 with a state-space method”
by Shinsuke Koyama, Taiki Horie and Shigeru Shinomoto

This article deals with the use of the Hawkes model to estimate breakpoints in the reproduction number R for the current COVID-19 pandemy. The subject is of course of importance, right now. The paper is really well written. The method is sound (despite some more minor things about bibliography mainly that I list below) and I think this article clearly deserves publication in PLOS computational biology. However the authors need maybe to go through some very recent papers that may help them to introduce their method with even more convincing arguments.

- First of all, at first glance, when one reads the paper, we think that the authors are using a point process approach based on a classical time continuous Hawkes model. In this respect the fact that one observes the counts day per day, makes the reader think that the authors are dealing with aggregated data. In this respect there is a very recent paper made by Cheysson and Lang

  `https://arxiv.org/abs/2003.04314`

  which explains how to deal with this discretization of the process that the authors might want to cite

- However, this is not what the authors are doing. It turns out that when reading the METHODS section, the authors are in fact using a discretized model, close to ARMA, but without a Gaussian noise and where the number of cases per day is a negative binomial. Moreover their bayesian model incorporates an autoregression on the reproduction number itself. All the arguments that the authors give for these choices are totally sound. I just feel that some of it might come sooner so that the reader is not mislead into thinking the authors use the classical Hawkes point process model, but a discretized variant of it.

- The authors are using a Cauchy noise in the autoregression of R_i, the reproduction number of day i (prior distribution). This choice is actually debated in the bayesian litterature, see

  `https://arxiv.org/pdf/1507.07170.pdf`

  Since the authors are using median and not mean of the posterior for the estimation of R_i, I think most of the critics might be resolved, but it might be worth testing a lighter tail for the prior (as in this paper) just to be sure that their result are robust to a change of prior.

- If I understood correctly, the authors have $R_i = max(0, X_i)$ where X_i satisfies the autoregression formula (6) with Cauchy noise. So for me, going from (8) to (10), more or less assume that the authors are neglecting the non linearity relationship between R_i and X_i. In this sense, I'm not totally sure that they are writing what they are doing in all generality, but maybe only what they are doing when X_i is far away from 0 (which is totally sound during the COVID epidemic). They should add a comment on this part.

- I have a small question (but it maybe difficult to answer in just this one paper). Do the authors think that they can estimate the impact of a total lockdown ? could they quantify it by grouping together all the countries that have used strong lockdown ?

- Finally, the authors are citing Chiang, Liu and Mohler [20] in their discussion. I would have cited them even sooner in the introduction. Chiang Liu and Mohler are clearly doing something totally different with the Hawkes model and despite the fact that both papers are using Hawkes for epidemic (as well as Cheysson and Lang for instance), the novelty of this present paper lies clearly in the modeling of the dynamic of the epidemic with respect to strong measures such as lockdown. And this evaluation cannot be done by any of the two other papers. I think a lot of people are right now investigating Hawkes processes and variants for epidemic modelling. What is the most important thing in my opinion, is this understanding in the dynamic of the reproduction number that the present authors provide.
