# Supplementary material for: Estimating the time-varying reproduction number of COVID-19 with a state-space method
Source: PLoS Comput Biol. 2021 Jan 29;17(1):e1008679. doi: 10.1371/journal.pcbi.1008679 (PMC7875393; doi:10.1371/journal.pcbi.1008679)
Supplement: Attachment — Submitted filename: Response to Reviewer.pdf [file pcbi.1008679.s002.pdf]

S. Koyama, T. Horie and S. Shinomoto
"Estimating the time-varying reproduction number of COVID-19 with a state-space method"

Dear Editor,

Thank you for handling our manuscript. We appreciate the reviewers' comments, which are constructive and important for improving our paper. We have revised the manuscript by taking account of all the comments. We attach here our point-by-point responses to the comments.

We feel that the manuscript was improved significantly and hope that it is now satisfactory. We look forward to hearing from you.

Best regards,

Shigeru Shinomoto
shinomoto.shigeru.6e@kyoto-u.ac.jp
Dept. Physics, Kyoto University, Kyoto, 606-8502, Japan

Reviewer #1

We would like to thank the reviewer for providing constructive comments. We have revised the manuscript by taking account of all the comments. Please find our point-by-point responses to the comments below.

Before going into individual responses, please note that we have reorganized the manuscript into the IMRAD (Introduction, Methods, Results, and Discussion) format because both reviewers recommended focusing on the methodological aspect. Also, we have updated the real data analysis by taking into account the newer data up to November $30th$. We believe that the new results may attract audiences.

Main comments

Comment *This is a really interesting paper, and I am glad more work is being added applying Hawkes processes to Infectious diseases. The method is very nice, and i think novel to my knowledge. I do like the embedding of epidemiological approaches in state space. There are however some issues which means their results can be hard to justify. The use of case data when not linking cases to infections means the authors do not consider any incubation period or the possibility of asymptomatic transmission. This dilutes the validity of their reproduction number estimates. They really need to either connect R→infection→cases. I could not see the choice of phi being discussed. I assume its exponential, but if so this is wrong for infectious diseases. Hawkes processes are fundamentally linked to this phi, without it the model is incomplete.*

Response We are glad that you found this paper interesting. Please note that we have explicitly introduced the distribution of transmission delays into the model; we have adopted a log-normal distribution with the mean 4.7 days and SD 2.9 days, which were estimated for the COVID-19 in the reference [12]. To make this point clearer, we added the following sentences and a new figure (Fig. 2) in the revised manuscript:

> " The distribution of transmission delays on a daily basis is given as the difference of a cumulative distribution function of the log-normal distribution, $\phi_d = \Phi_d - \Phi_{d-1}$, where
>
> $$\Phi_d = \frac{1}{2}\mathrm{erfc}\left(-\frac{\log d - \mu}{\sqrt{2\sigma^2}}\right), \quad (1)$$
>
> where the parameters μ and σ are given in terms of the mean $m = 4.7$ and the SD $s = 2.9$ as $\mu = \log(m^2/\sqrt{s^2+m^2})$ and $\sigma = \sqrt{\log(1+s^2/m^2)}$ (Fig. 2). " (page 4)

Comment *I do comment the authors on their method, aside from a few missed details it is interesting and novel, but the could do much more to make their model more useful and accurate by considering the epidemiology. So the authors are best either focusing on a methods paper, and in which case a comparison to existing approaches (in addition to WT) would be well received, and of-course heavy caveating of the results. As an application paper, i would like a more careful consideration of the data.*

Response According to your suggestion, we reorganized the manuscript into the IMRAD format, emphasizing the methodological aspect. We also compared our method with "EpiEstim" proposed by Cori et al. [28], in addition to the WT method. Accordingly, we rewrote the paragraph on the comparison study as follows:

> " We compared our method with the following two conventional estimation methods in their ability at estimating the time-varying reproduction number of the synthetic data. A method suggested by Wallinga and Teunis (WT method) estimates the "case reproduction number" [25, 26],
>
> $$R_i = \sum_{j=i+1}^{T} \frac{\nu_j \phi_{j-i}}{\sum_{k=1}^{j-1} \nu_k \phi_{j-k}}. \tag{2}$$
>
> Another method suggested by Cori et al. (EpiEstim) estimates the "instantaneous reproduction number" [27, 28],
>
> $$R_i = \frac{\nu_i}{\sum_{j=1}^{i-1} \nu_j \phi_{i-j}}, \tag{3}$$
>
> in a Bayesian framework with a Poisson likelihood and a gamma-distributed prior for R_i. We plotted the estimation results obtained by the WT method and EpiEstim with a sliding window of 7 days in Fig. 3. It is observed that both methods are easily influenced by the fluctuation of data and accordingly it is difficult to discern the change points in the original process. Furthermore, the WT method tends to underestimate the current reproduction number at the end of the recorded interval, because it requires data that could be obtained in the future; the reproduction number estimated by EpiEstim is shifted backward in time relative to the WT and our methods because it uses only data from time points before i. " (page 7)

Comment *I hope the authors will not begruge me, I realise these revisions would require quite a bit more work, but I believe they will strengthen the paper and ultimately help people using this in the future. However in general, I am very supportive of this work and belive it to be a good addition to the literature.*

Response We appreciate your support. We did our best to revise our manuscript by taking into account all your suggestions.

Other comments

1) *We have applied the state-space method ← this is introduced without me as the reader knowing what the authors are referring to*

Response We added the following sentence at the beginning of the Methods section to describe the state-space method briefly with a reference to the standard textbook by Durbin and Koopman:

> " The state-space method describes an evolution of a system by a set of first-order difference equations of state variables. The state variables can be inferred from measured data using a recursive Bayesian estimation [16]. " (page 3)

2) *Limitations of using World in data should be added, factors like reporting accuracy and delay etc*

Response We have discussed the limitations of the data and the way of dealing with them in the discussion section:

> " When inferring the transmission of disease from daily confirmed cases, we have considered potentially erroneous observations made in the real data. We took into account counting errors by assuming a negative binomial distribution that represents the over-dispersion. We also took into account the variation by day of the week and adjusted the data by compensating for the periodic dependency. Note that there may still be an underestimation of infection numbers, as asymptomatic cases may have been overlooked. Though this is unavoidable unless the inspection is enforced, it is reported that the infections caused by asymptomatic people are relatively small (about 6%) for COVID-19 [19]. " (page 11)

3) *It is interesting to note that the drop in the reproduction number occurred after political measures, such as lockdown and border closure, were enforced. ← A few points about why this might be the case would be good. Factors like slow adherence, but more plausibly that cases are lagged ahead of infections by an incubation period of 5 days or so. Obviously reporting is the biggest issue, testing was poor in most of europe in April.*

Response We discussed this point in the discussion section:

> " Interestingly, the drop in the reproduction number occurred after political measures, such as lockdown and border closure. It should be also noted that there may be an additional latency between the times at which political measures were conducted and the changes in the reproduction number, which may

reflect the behavior change. This delay may also be country-specific. Therefore, it could be interesting to investigate the delay in the change-points in the reproduction number following social events. " (page 11)

4) *A discussion of why the reproduction numbers differ at the start is good but i would like the authors to consider if its their modeling assumptions or data censoring rather than real effects.*

Response By following the comment, we added a paragraph in the discussion "Pros and cons" concerning the effect of data censoring on the estimates of the reproduction number:

> " Also, we did not address censoring for incomplete observation of the epidemic process in particular at the initial stage. These may cause bias in the estimations of the reproduction number at the early stage of the epidemic. " (page 11)

5) *The choice of kernel needs to be fully described in the main text. it underpins most of the dynamics*

Response We added sentences (on page 4) and a new figure (Fig. 2) to describe the kernel in detail. (Please see our response to your main comment.)

6) *The sensitivity analysis of lambda should be presented in this paper*

Response We tested different values of the hyperparameter γ of the Cauchy distribution and confirmed that the estimate of λ_i is robust against the value of γ. Accordingly, we inserted the following sentences in the revised manuscript:

> " We tested different values for the hyperparameter γ as 10^{-2}, 10^{-3}, and 10^{-4}, and observed that the estimated reproduction number $\hat{R}_i$ is sensitive to the value of γ while the estimated total rate $\hat{\lambda}_i$ is robust against γ (results not shown). " (page 6-7)

7) *Posterior convergence and stats should be provided in the paper*

Response We performed the sequential Monte Carlo analyses using different numbers of particles, 10^3, 10^4, 10^5 and 10^6 and examined the convergence in terms of the posterior estimates of $\hat{R}_i$. We confirmed that the standard deviation of $\hat{R}_i$ decreases reasonably with the number of particles. Accordingly, we added the following paragraph and a new table in the revised manuscript:

“ To verify the convergence of the posterior estimate of $\hat{R}_i$ concerning the number of particles, we computed the standard error of $\hat{R}_i$ with 100 cases of the Monte Carlo estimation (Table I). We observed that 10^6 particles may provide a reasonably accurate estimate of the reproduction number.” (page 6)

particles	10^3	10^4	10^5	10^6
standard error	0.165	0.057	0.028	0.010

Table 1: The standard error of $\hat{R}_i$ computed with 100 cases of the sequential Monte Carlo algorithm applied to the synthetic data in Fig. 3a.

8) *Why is L 30*

Response We have chosen $L = 30$ because this is large enough to approximate the original rate process, as the transmission delay kernel ϕ_d is negligible at $d = 30$. We inserted the following sentence in the revised manuscript:

> “ We have chosen $L = 30$ in the following analysis because the transmission delay kernel ϕ_d is negligible at $d = 30$ (Fig. 2). ”
>
> (page 5)

9) *The choice of spontaneous occurrences wrong, importation happened at least for part of the period this paper considers*

Response Thank you for pointing this out. We agree with your comment. We inserted the following discussion in the revised manuscript:

> “ Here, we set the spontaneous occurrence rate to zero ($\mu' = 0$) in the analysis of real data. However, imported cases might be involved in the data. Also, we did not address censoring for incomplete observation of the epidemic process in particular at the initial stage. These may cause bias in the estimations of the reproduction number at the early stage of the epidemic. ” (page 11)

10) *What is phi*

Response ϕ_d is the transmission delay kernel, which is set to a log-normal distribution with mean 4.7 days and SD 2.9 days. We added a detailed description of ϕ_d in the revised manuscript. Please see our response to your main comments above.

Minor comments

1) *The Kernel definition in (2) is nonstandard for a Hawkes process and can lead to the impression that its a discrete model*

Response We did not use the original Hawkes process but employed a discrete variant. We rewrote the manuscript to avoid confusion.

2) *I do like the decomposition of the kernel into R and events, its novel*

Response Thank you. We are glad that you found it novel.

3) *Might be worth mentioning the US for type C*

Response By updating the real data analysis up to November $30th$, we have seen that all countries have different dynamics and they cannot be classified into three types. Accordingly, we described the time course of new cases for each country, instead of categorizing the countries into three classes. (page 8)

4) *The authors might consider looking at the HawkesN variant - they will find its like to SIR models interesting*

Response Thank you for letting us know about the interesting work done by Rizoiu et al. (2018). We read their paper carefully and found that although they established a link between SIR and Hawkes models, it is valid only when the exponential kernel is assumed. The advantage of the Hawkes process over SIR is that it allows us to use any kernel for delay distribution. We discussed this point in the introduction:

> " Though ODE models also assume the transmission delay, such that the SIR model represents the situation in which delays are distributed exponentially [8], they cannot adopt the specific distribution of delays for each disease. " (page 2)

Reviewer #2

We would like to thank the reviewer for providing constructive comments. We have revised the manuscript by taking account of all the comments. Please find our point-by-point responses to the comments below.

Before going into individual responses, please note that we have reorganized the manuscript into the IMRAD (Introduction, Methods, Results, and Discussion) format because both reviewers recommended focusing on the methodological aspect. Also, we have updated the real data analysis by taking into account the newer data up to November $30th$. We believe that the new results may attract audiences.

1) *First of all, at first glance, when one reads the paper, we think that the authors are using a point process approach based on a classical time continuous Hawkes model. In this respect the fact that one observes the counts day per day, makes the reader think that the authors are dealing with aggregated data. In this respect there is a very recent paper made by Cheysson and Lang*
`https://arxiv.org/abs/2003.04314`
which explains how to deal with this discretization of the process that the authors might want to cite

Response Thank you for letting us know about the interesting paper by Cheysson and Lang. We added the following paragraph in which we refer to their paper and discuss the difference between our method and their approach to deal with counts data:

> " We have converted the original Hawkes process Eq. (1) into a discrete-time variant representing the expected number of events on a daily basis Eq. (2) because the exact timing of infection event is not available for the case of COVID-19. It is noteworthy that Cheysson and Lang also developed a method for estimating parameters of the Hawkes process from counts data [30]. However, their method is based on a spectral likelihood, assuming stationarity in the underlying process. In contrast to this, we directly modeled the count time series and combined it with the state-space model to accommodate nonstationary data. " (page 10-11)

2) *However, this is not what the authors are doing. It turns out that when reading the METHODS section, the authors are in fact using a discretized model, close to ARMA, but without a Gaussian noise and where the number of cases per day is a negative binomial. Moreover their bayesian model incorporates an autoregression on the reproduction number itself. All the arguments that the authors give for these choices are totally sound. I just feel that some of it might come sooner so that the reader is not mislead into thinking the authors use the classical Hawkes point process model, but a discretized variant of it.*

Response Yes, we used a discretized model, not the original Hawkes process as you pointed out. We rewrote our manuscript to avoid confusion. Also, please see our response to your comment 1).

3) *The authors are using a Cauchy noise in the autoregression of R_i, the reproduction number of day i (prior distribution). This choice is actually debated in the bayesian litterature, see*
https://arxiv.org/pdf/1507.07170.pdf
Since the authors are using median and not mean of the posterior for the estimation of R_i, I think most of the critics might be resolved, but it might be worth testing a lighter tail for the prior (as in this paper) just to be sure that their result are robust to a change of prior.

Response In the state-space methods, it is the key to use a heavy-tailed distribution, such as the Cauchy distribution, for the system to infer stepwise changes, as discussed in Kitagawa (1987). We added the following paragraph to explain it as well as a drawback of using the Cauchy distribution by referring to the paper you mentioned:

> " We introduced the Cauchy distribution, Eq. (6), into our analysis, assuming the stepwise changes in the reproduction number R_i. Accordingly, we were able to infer change-points from the posterior distribution taking on stepwise characteristics. As discussed in [20], the use of the heavy-tailed distribution enables us to detect change-points; using a Gaussian noise, instead of the Cauchy noise, results in gentle changes in the reproduction number. However, a drawback of the Cauchy distribution is that it causes slow convergence in the Monte Carlo simulation [31]. " (page 11)

4) *If I understood correctly, the authors have $R_i = \max(0, X_i)$ where X_i satisfies the autoregression formula (6) with Cauchy noise. So for me, going from (8) to (10), more or less assume that the authors are neglecting the non linearity relationship between R_i and X_i. In this sense, I'm not totally sure that they are writing what they are doing in all generality, but maybe only what they are doing when X_i is far away from 0 (which is totally sound during the COVID epidemic). They should add a comment on this part.*

Response We take the nonlinear relationship between R_i and x_i via a ramp function $R_i = f(x_i) = \max(0, x_i)$ in the state space form as well. To express it more explicitly, we rewrote the equation (7) in page 4 (equation (8) in the original paper) as

$$\lambda_j = \mu' + \sum_{i=j-L}^{j-1} \nu_i f(x_i) \phi_{j-i}$$

5) *I have a small question (but it maybe difficult to answer in just this one paper). Do the authors think that they can estimate the impact of a total lockdown ? could they quantify it by grouping together all the countries that have used strong lockdown ?*

Response Thank you for your stimulating comment. Though the problem is beyond the scope of our manuscript, we are inspired to make the following analysis. Because the lockdown may cause a decrease in the reproduction number, the minimum reproduction number achieved so far may represent the impact of lockdown in each country. We added the following subsection to address this question:

> " The degree of a drop in the estimated reproduction number could reflect the impact of non-pharmaceutical interventions such as a lockdown. It might be possible to quantify the effectiveness of political interventions in each country in terms of the relative percentage reduction in the reproduction number [12]. Here we searched for the minimum reproduction number averaged over 10 days that was achieved in each country. Figure 7a depicts the reproduction number for 10 days whose average takes minimum in each country. The variation in the numbers of daily new cases is depicted in Fig. 7b, indicating that the estimated reproduction number is correlated to the slope in the log plot.
>
> " (page 9)

6) *Finally, the authors are citing Chiang, Liu and Mohler [20] in their discussion. I would have cited them even sooner in the introduction. Chiang Liu and Mohler are clearly doing something totally different with the Hawkes model and despite the fact that both papers are using Hawkes for epidemic (as well as Cheysson and Lang for instance), the novelty of this present paper lies clearly in the modeling of the dynamic of the epidemic with respect to strong measures such as lockdown. And this evaluation cannot be done by any of the two other papers. I think a lot of people are right now investigating Hawkes processes and variants for epidemic modelling. What is the most important thing in my opinion, is this understanding in the dynamic of the reproduction number that the present authors provide.*

Response By following your advice, we cited Chiang, Liu, and Mohler in the introduction (page 2)

> " Recently, Chiang, Liu, and Mohler [13] modeled COVID-19 transmission using the Hawkes process [14], in which the delay distribution can be explicitly adopted as a self-exciting kernel. They combined the Hawkes process with spatial and temporal covariates, such as demographic features and Google mobility indices, to explain the variability of the reproduction number, and to forecast future cases and deaths in the USA. "

As in our response to your first comment, we addressed Cheysson and Lang's work and discussed the difference between our method and their approach. (Please see our response to your first comment.)
